# The Impact of Preoperative Radiomics Signature on the Survival of Breast Cancer Patients With Residual Tumors After NAC

**DOI:** 10.3389/fonc.2020.523327

**Published:** 2021-02-03

**Authors:** Ling Zhang, Xinhua Jiang, Xiaoming Xie, Yaopan Wu, Shaoquan Zheng, Wenwen Tian, Xinhua Xie, Li Li

**Affiliations:** ^1^ State Key Laboratory of Oncology in South China, Department of Radiology, Sun Yat-sen University Cancer Center, Collaborative Innovation Center for Cancer Medicine, Guangzhou, China; ^2^ State Key Laboratory of Oncology in South China, Department of Breast Surgery, Sun Yat-sen University Cancer Center, Collaborative Innovation Center for Cancer Medicine, Guangzhou, China

**Keywords:** breast cancer, neoadjuvant chemotherapy, radiomics, prognosis, nomogram

## Abstract

**Background:**

Residual cancer cells remaining after chemotherapy may have more aggressive behavior that promotes recurrence or metastasis, and which patients would benefit from subsequent additional treatment is controversial. The purpose of our study was to evaluate the prognostic value of the preoperative radiomics features of computed tomography (CT) imaging in breast cancer (BC) patients with residual tumors after neoadjuvant chemotherapy (NAC).

**Methods:**

Post-NAC CT images were reviewed from 114 patients who had received breast surgery and had residual breast tumors. The association of the 110 radiomics features derived from CT images with 5-year disease-free survival (DFS) was assessed by log-rank test in the training cohort, resulting in 13 prognostic radiomics features.

**Results:**

We constructed a radiomics signature consisting of four selected features by using least absolute shrinkage and selection operator (LASSO) Cox regression analysis, which performed well in the discrimination with an area under the curve (AUC) of 0.78 (95% CI, 0.67–0.89) and 0.73 (95% CI, 0.59–0.87) in the training and validation cohorts, respectively. Radiomics nomogram, incorporating the radiomics signature with the conventional clinical variables, also performed well in the two cohorts (training cohort: AUC, 0.84; validation cohort: AUC, 0.82). Moreover, we found that the high-risk patients determined by our radiomics nomogram could benefit from postoperative adjuvant chemotherapy, while the low-risk and total patient groups could not.

**Conclusions:**

Our novel radiomics nomogram is a promising and favorable prognostic biomarker for preoperatively predicting survival outcomes and may aid in clinical decision-making in BC patients with residual tumors after NAC.

## Introduction

Neoadjuvant chemotherapy (NAC) in the management of breast cancer (BC) has become a popular treatment strategy in recent years (1, 2). Only a subset of patients will achieve a pathological complete response (pCR) following NAC, defined as absence of invasive cancer in the breast and the axillary lymph nodes (ALNs), with rates varying according to the different subtypes of BC (3, 4). The presence of residual tumor following NAC indicates the increased recurrence risk; however, to date, the role of additional postoperative adjuvant chemotherapy for non-pCR patients is not clear, although non-pCR is clearly associated with high recurrence and metastasis (4–6). Therefore, a more accurate understanding of the molecular and genomic characteristics of tumors will undoubtedly facilitate the development of clinical trials for the treatment of residual diseases (3, 7, 8). Moreover, identifying high-risk, non-pCR patients using noninvasive approaches for additional treatments is urgently needed.

There is evidence that radiogenomics can define the association between imaging features and genomic phenotypes, which has recently attracted great interest (9, 10). To facilitate the use of image features to directly estimate patients’ outcomes, "radiomics" has made rapid progress (11). It is now possible to extract quantitative risk variables from traditional computed tomography (CT) images to achieve non-invasive profiling of tumor heterogeneity (11, 12). To date, radiomics has made great contributions in the field of cancer and has been widely applied to tumor detection, subgroup identification, treatment response evaluation, and so on. A multiple radiomics features-based signature is often more valuable than a single biomarker, and a recent study has shown that radiomics features from magnetic resonance imaging (MRI) performed well in the prognostic prediction of BC (13–15). However, to our knowledge, CT images radiomics features-based signatures have not yet been deeply assessed, especially in non-pCR BC patients after NAC.

Therefore, the purpose of this study is to develop and validate a CT-based radiomics signature and nomogram to predict the 5-year disease-free survival (DFS) and response of additional chemotherapy in non-pCR BC patients and then to precisely guide the implementation of postoperative adjuvant chemotherapy.

## Materials and Methods

### Patients

Ethical approval for this retrospective study was granted by the ethics committee of Sun Yat-sen University Cancer Center. Four hundred sixty-two consecutive invasive breast cancer patients (mean age, 49 years) who received neoadjuvant chemotherapy (NAC) before surgery between January 2010 and December 2016 were identified. All patients were treated at the Sun Yat-sen University Cancer Center, and the corresponding ethical approval was obtained for this retrospective analysis at our cancer center. The informed consent requirement was waived due to the retrospective nature of the study. The inclusion criteria of our study were as follows: (a) preoperative dynamic contrast enhanced chest CT performed <30 days before surgical resection at our institution; (b) initial unilateral breast malignancy with histologically confirmed invasive breast cancer; (c) residual breast tumor after NAC; (d) a lesion presenting as a mass on CT; (e) no other malignant neoplasm found previously; (f) available clinicopathological characteristics and follow-up data. The exclusion criteria of our study were as follows: (a) tumor lesions that could not be recognized by CT; (b) patients with distant metastatic disease after six- or eight-cycle preoperative chemotherapy; (c) CT images of poor quality and with large artifacts, which cannot therefore be used for analysis. Finally, 114 female patients (mean age, 48 years; range, 30–69 years) were included in this study (**Figure S1**).

### Clinical Factors and Follow-Up

The potential DFS-related clinical risk factors of the enrolled patients were collected, including age, menopausal status, tumor grade, vascular invasion, estrogen receptor (ER) status, progesterone receptor (PR) status, human epidermal growth factor receptor 2 (HER2) status, Ki-67 expression level, adjuvant treatment after surgery, and so on. Invasive tumors with HER2 scores of 3+ by immunohistochemistry (IHC) were defined as positive, while fluorescence *in situ* hybridization (FISH) was conducted to determine HER2 amplification for tumors with HER2 scores of 2+ by IHC (**Table S1**). The end point of our study was DFS, which was determined as the time from the date of surgery to the date of relapse (event), death, or from the date of surgery to the date that the patient was last known to be free of relapse or death (censored). All enrolled patients had been followed up for at least 3 months after surgery.

### CT Image Acquisition and Preparation for Radiomics Analysis

**Figure 1:**
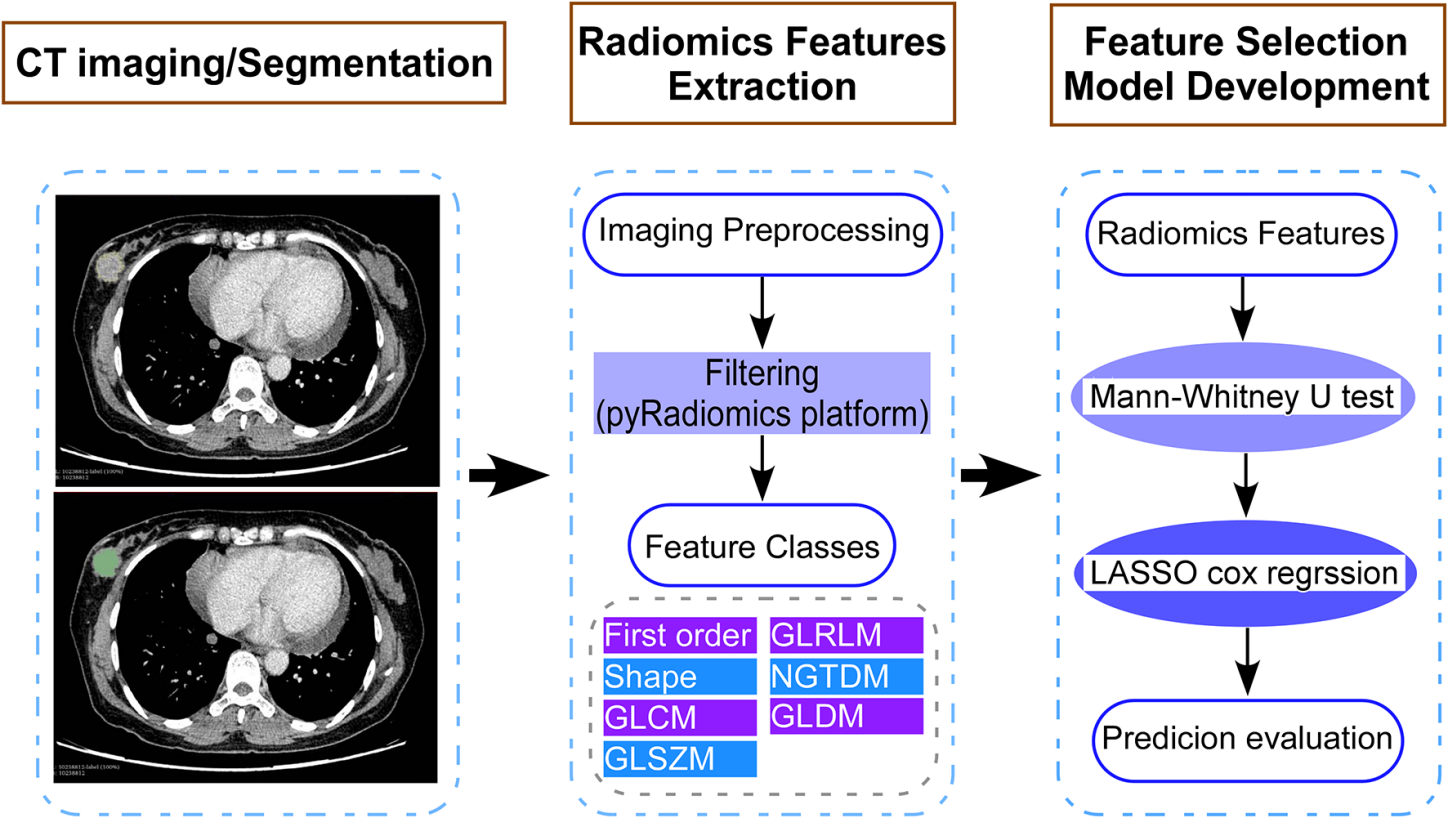
Study flow diagram showing the development of a computed tomography (CT) imaging-based model for patients with breast cancer who had residual tumors after neoadjuvant chemotherapy. The steps include (1) CT image acquisition and segmentation, (2) extraction of features using the pyradiomics platform, and (3) selection of CT image features and construction of the model. LASSO, least absolute shrinkage and selector operation.

All patients underwent contrast-enhanced CT. The radiomics workflow is shown in **Figure 1**. Before receiving breast-conserving surgery or mastectomy after NAC, all patients underwent contrast-enhanced chest CT with a 64-slice spiral CT scanner (64-slice CT750 HD scan, GE Medical Systems). The acquisition parameters of the CT scan were as follows: 120 kV, 200 effective mAs, a rotation time of 0.4 or 0.5 s, detector collimation of 64 × 0.625 mm, a matrix of 512 × 512, and a thin layer reconstruction layer thickness of 1.25 mm. After conventional nonenhanced CT scanning, 85–100 ml of contrast agent (iopamidol, 300 mg i/ml, Bracco) was intravenously administered, and dynamically contrast-enhanced CT scans were performed at a speed of 3.5 ml/s, followed by 30 ml saline flushing. Arterial- and vein-phase images were obtained at 30 and 60 s, respectively. Tumor regions of interest (ROI) were semi-automatically segmented in vein-phase images three-dimensionally using 3D Slicer software. Radiomics features were extracted from enhanced CT images by using pyradiomics. Intraclass correlation coefficients (ICCs) were used to evaluate the repeatability of radiomics features extraction within and among observers. Two experienced radiologists analyzed the repeatability between observers for determining ROI segmentation-based radiomics features (readers 1 and 2 with 5 and 8 years of clinical imaging reading experience in chest CT), reader1 and reader 2 repeated features extraction independently on 30 randomly chosen patients. The radiologists did not know anything about the clinical or pathological data of patients but were told that the patients had breast carcinoma.

### Development and Validation of the CT-Based Radiomics Signature and Nomogram

Radiomics features were extracted from the tumor regions of interest (ROIs) drawn from CT images by the radiologists. The features were computed using the pyradiomics package from the Python platform. In total, 110 features in seven distinct categories were extracted. The features were grouped into First Order Statistics (19 features), Shape-based (16 features), Gray Level Cooccurence Matrix (24 features), Gray Level Run Length Matrix (16 features), Gray Level Size Zone Matrix (16 features), Neighboring Gray Tone Difference Matrix (5 features), and Gray Level Dependence Matrix (14 features). We randomly divided patients into two groups: a training cohort (n = 76) and a validation cohort (n = 38). The characteristics of the patients in the training cohort and the validation cohort were compared by variance for continuous variables and chi-squared test or Fisher’s exact test for categorical variables. In multivariate analysis, the number of events should be at least 10-fold larger than the number of covariates included according to the Harrell guideline. To solve this problem in high-dimensional data, we conducted least absolute shrinkage and selection operator (LASSO) Cox regression analysis to choose the most useful prognostic radiomics features in the training data cohort. The radiomics signature was constructed based on the selected imaging features, and the risk score of each patient was calculated using a linear combination of the selected features weighted by their respective coefficients. Among the enrolled patients, univariate Cox regression analysis was first performed to screen DFS-associated CT image-based features (all features were standardized using Z-score) in the training cohort and then validated in the validation cohort. The patients were then classified into high- or low-risk groups according to the radiomics signature, the threshold of which was confirmed with receiver operating characteristic (ROC) curve analysis. Kaplan-Meier curves and log-rank tests were used to compare survival between the high- and low-risk groups. In the training cohort, a univariate Cox proportional hazards model was used to verify the effects of clinicopathological variables (age, menopausal status, initial tumor status, initial node status, initial ER status, initial PR status, initial HER2 status, initial Ki-67 expression level, tumor size at surgery, grade at surgery, vascular invasion at surgery, ALN status at surgery, ER status at surgery, PR status at surgery, HER2 status at surgery, Ki-67 expression at surgery, adjuvant chemotherapy, and adjuvant endocrine therapy) and the radiomics signature on DFS. Variables determined as significant in the univariate Cox proportional hazard model (P < 0.05) were included in the multivariate Cox proportional hazard model. To prove the value of the radiomics signature, a radiomics nomogram was developed in the training cohort and then evaluated in the validation cohort. The radiomics nomogram combined the radiomics signature and various clinical risk factors based on the multivariate Cox analysis with stepwise selection. The performance of the radiomics nomogram was analyzed by calibration curves. The area under the curve (AUC) between the predicted probability and the actual result was computed to evaluate the predictive ability and discriminability of the model (1.0 indicates a perfect discrimination; 0.5 indicates no better discrimination than random chance). Decision curve analysis (DCA) was also used to evaluate the clinical usefulness of the radiomics nomogram by quantifying the net benefits at different threshold probabilities.

### Statistical Analysis

All statistical analyses were conducted with R statistical software (version 3.5.3; https://www.r-project.org/). The R packages used in our study were as follows: “glmnet,” “rms,” “Hmisc,” “pROC,” “survival,” and “dca.R.” The LASSO Cox regression analysis was applied with L1-penalty parameter tuning (λ), performed by 10-fold cross-validation based on the minimum criteria. A conventional two-tailed P value <0.05 was determined to be significant.

## Results

### Clinical Characteristics

A total of 114 patients who underwent breast-conserving surgery or mastectomy after NAC were included in our study. There were 36 events (10 local-regional recurrence, 4 contralateral breast, and 22 distant metastasis) during a mean follow-up period of 44.4 months (range, 5–93 months). The mean time to disease event was 21.2 months (range, 5–78 months). Disease events occurred in three patients during a follow-up period of the first 6 months, which might have resulted from residual disease. The patients' clinical characteristics are summarized in **Table S1**. The baseline clinical characteristics of patients were similar among the two cohorts (P < 0.05). Among patients enrolled, 37.7% were treated with postoperation chemotherapy, while all patients received adjuvant radiotherapy. The intraobserver agreement of the radiomics features extraction between the two readers was excellent [the mean ICC value was 0.982 (range, 0.781–0.999)]. Therefore, all the results are based on the first radiologist's measurements.

### Development of Radiomics Signature-Based Model

**Figure 2:**
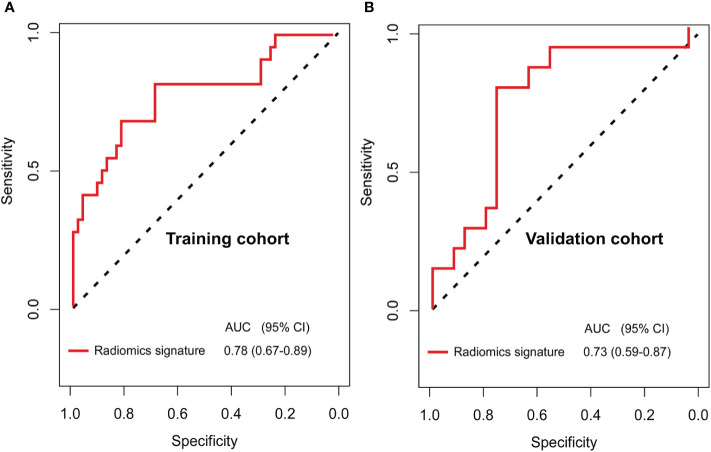
Receiver operating characteristic (ROC) curves for the newly developed computed tomography (CT) imaging features-based radiomics signature **(A, B)** in the training (AUC, 0.84) and validation (AUC, 0.82) cohorts of patients with breast cancer who have residual tumors after neoadjuvant chemotherapy.

**Figure 3:**
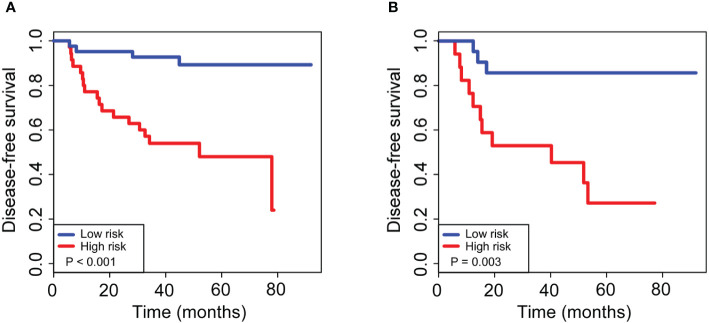
Kaplan–Meier curves for 5-year disease-free survival in breast cancer patients who had residual tumors after neoadjuvant chemotherapy. For patients with high and low risks of DFS, stratified by our newly developed radiomics signature, the 5-year survival rates were 89 and 86% for the low-risk group and 48 and 27% for the high-risk group, with a significant difference between groups (P < 0.01) in both the training **(A)** and validation **(B)** cohorts. The log-rank test was used to calculate the corresponding P-values.

The 110 radiomics features were extracted from the CT images of enrolled patients. From these candidate features, we selected 13 potential DFS-associated predictors from the 110 features identified in the training cohort using univariate Cox proportional regression analysis. In addition, we conducted colinear analysis and discovered colinearity in some predictors, which may affect the accuracy of the traditional Cox regression analysis (**Figure S2**). To minimize the colinearity between variables, we applied a Cox regression model combined with the LASSO algorithm to further eliminate nine features, yielding a final four-feature panel (**Figure S3**). We then calculated the risk score of the radiomics signature for every BC patient based on the values of the final four remaining features weighted by their regression coefficients: risk score = original_firstorder_Kurtosis*(−5.570173e-03)+original_glcm_Correlation*(1.004042e+00)+original_glcm_MaximumProbability*(−9.648413e-01)+original_glszm_LargeAreaEmphasis*(1.643472e-08). The radiomics signature showed an AUC of 0.78 (95% CI, 0.67–0.89) in the training cohort and 0.73 (95% CI, 0.59–0.87) in the validation cohort (**Figures 2A, B**). The optimum cutoff value was 0.185 generated by the ROC curve analysis. Patients were stratified into a low-risk group (radiomics signature <0.185) and a high-risk group (radiomics signature ≥0.185) based on this cutoff value. Furthermore, Kaplan-Meier curves showed statistically significant difference between the two groups in the training (P < 0.001) and validation cohorts (P = 0.003) (**Figures 3A, B**).

### Performance and Validation of the Radiomics Nomogram for Individualized Prediction

In the training cohort, the results of the univariate analysis based on the training cohort are shown in **Table 1**. The radiomics signature, Ki-67 expression at surgery and ALN status at surgery were associated with DFS. In the multivariate Cox regression analysis with stepwise selection, the radiomics signature (DFS hazard ratio, 30.328; 95% CI, 2.677–343.550; P = 0.006), Ki-67 expression at surgery (DFS hazard ratio, 1.018; 95% CI, 1.001–1.035; P = 0.041), and ALN status at surgery (DFS hazard ratio, 2.360; 95% CI, 1.032–5.397; P = 0.042) remained independent prognostic features in the final Cox proportional hazards model (**Table 2**).

**Table 1:** Univariate cox analysis of disease-free survival in the training cohort.

Variable	Hazard ratio	95% CI	*P*-value
Age, mean ± SD, years	0.985	0.940–1.031	0.512
Menopausal status			
Pre	Ref		
Post	0.992	0.417–2.373	0.992
Initial tumor status			0.381
T2	Ref		
T3	0.333	0.075–1.478	0.148
T4	1.053	0.419–2.648	0.912
Initial node status			
Negative	Ref		
Positive	24.082	0.068–8518.984	0.288
Initial ER status			
Negative	Ref		
Positive	1.276	0.533–3.057	0.585
Initial PR status			
Negative	Ref		
Positive	0.553	0.238–1.286	0.169
Initial HER-2 status			
Negative	Ref		
Positive	0.660	0.277–1.575	0.349
Initial Ki-67 (%), mean ± SD	1.021	0.999–1.044	0.060
Tumor size at surgery			0.868
≤2 cm	Ref		
2–5 cm	0.908	0.362–2.273	0.836
>5 cm	1.044	0.276–3.944	0.949
Grade at surgery			
I/II	Ref		
III	0.931	0.275–3.150	0.909
Vascular invasion at surgery			
Absent	Ref		
Present	0.452	0.103–1.974	0.291
ALN status at surgery	3.716	1.665–8.293	0.001
ER status at surgery			
Negative	Ref		
Positive	0.757	0.322–1.781	0.524
PR status at surgery			
Negative	Ref		
Positive	0.557	0.232–1.333	0.188
HER-2 status at surgery			
Negative	Ref		
Positive	0.908	0.392–2.101	0.821
Ki-67 at surgery (%), mean ± SD	1.023	1.008–1.038	0.002
Adjuvant chemotherapy			0.370
No	Ref		
Yes	0.473	0.173–1.290	0.144
Adjuvant endocrine therapy			
No	Ref		
Yes	1.500	0.638–3.525	0.352
Radiomics signature	109.301	11.885–1005.233	<0.001

ALN, axillary lymph node.

**Table 2:** Multivariate cox regression analysis for DFS in NAC-BC.

Variable	Hazard ratio (95% CI)	*P*-value
Ki-67 at surgery	1.018 (1.001–1.035)	0.041
ALN status at surgery	2.360 (1.032–5.397)	0.042
Radiomics signature	30.328 (2.677–343.550)	0.006

ALN, axillary lymph node.

**Figure 4:**
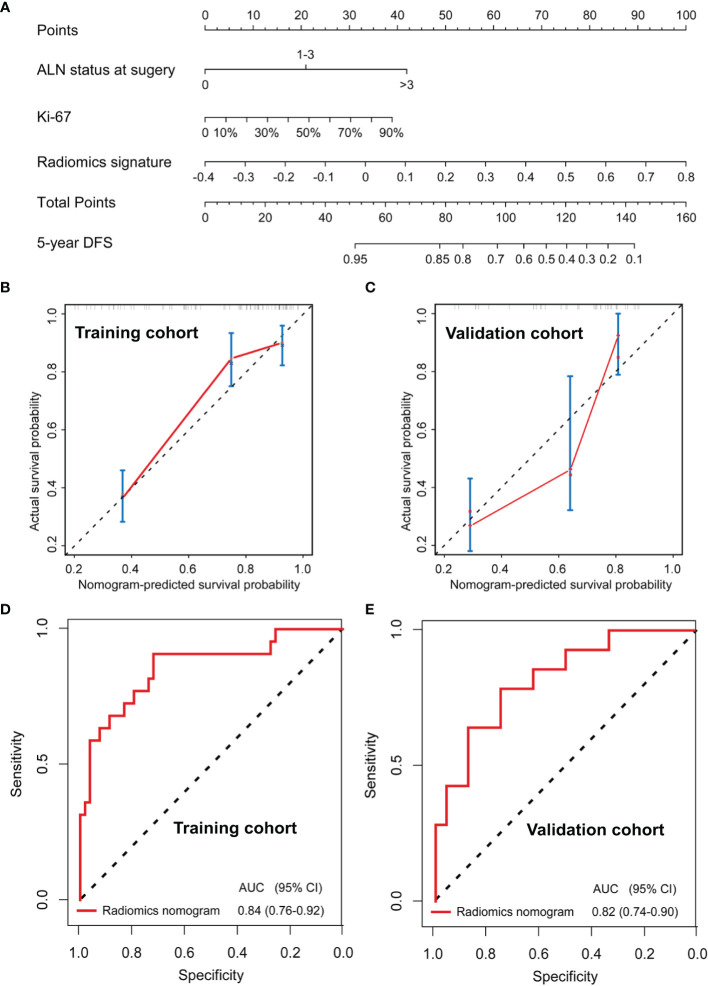
Nomogram for the prediction of disease-free survival (DFS) in breast cancer patients who had residual tumors after neoadjuvant chemotherapy **(A)**. Calibration curves of the nomograms developed in the training **(B)** and validation **(C)** cohorts. The ROC curves of the radiomics nomogram in the training (AUC, 0.84) and validation (AUC, 0.82) cohorts **(D, E)**.

**Figure 5:**
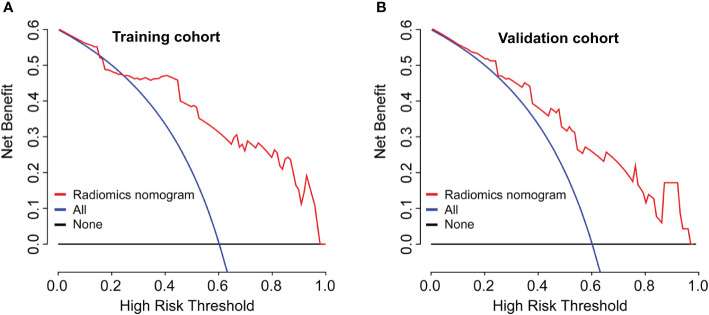
Model comparisons and clinical usefulness of the radiomics nomogram. Decision curve analysis of the prediction nomogram model in the **(A)** training cohort and **(B)** validation cohort. The solid black line represents the net benefit when no breast cancer patients are thought to have relapse or death, while the solid blue line indicates the net benefit when all patients with breast cancer are thought to have relapse or death. The solid red line indicates the net benefit when all breast cancer patients are considered based on our newly constructed radiomics nomogram model. In this study, the decision curves show that the radiomics nomogram predicting DFS adds more benefit than treating either all or no patients if the threshold probability is not between 19 and 27% in both the training and validation cohorts, while the ideal model refers to the model with the highest net benefit at any given threshold.

A radiomics nomogram was developed based on the radiomics signature and clinical risk factors (Ki-67 expression at surgery and ALN status at surgery) for predicting the risk of disease in patients with non-pCR BC (**Figure 4A**). Calibration curves (**Figures 4B, C**) showed good performance in the training and validation cohorts. Compared to the radiomics signature, the radiomics nomogram showed a better discrimination performance in the training (AUC, 0.84; 95% CI, 0.76–0.92) and validation cohorts (AUC, 0.82; 95% CI, 0.74–0.90) (**Figures 4D, E**). The DCA indicated that when the threshold probability for a patient was not between 19 and 27%, the nomogram showed better net benefit than the “treat all” or “treat none” strategy. The DCA for the nomogram is presented in **Figure 5**.

### Association With Additional Chemotherapy and Clinical Outcome

**Figure 6:**
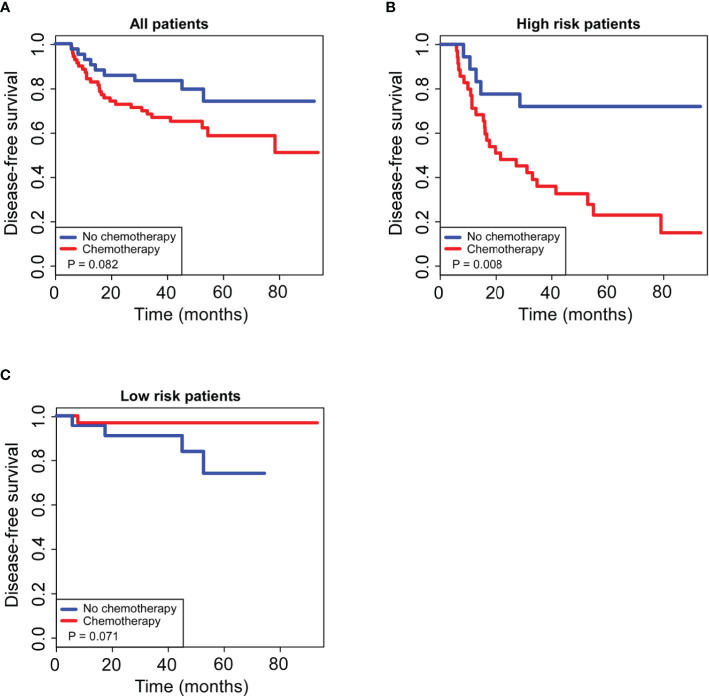
Kaplan–Meier curves of postoperative adjuvant chemotherapy for 5-year survival in non-pCR patients. For all patients enrolled in our study **(A)**, postoperative adjuvant chemotherapy had no significant effect on 5-year DFS (P > 0.05); for patients in the low-risk group stratified by the radiomics nomogram **(B)**, postoperative adjuvant chemotherapy had no significant effect on 5-year DFS (P > 0.05); for patients in the high-risk group stratified by the radiomics nomogram **(C)**, postoperative adjuvant chemotherapy significantly improved 5-year DFS (P < 0.05).

For all total patients, postoperative chemotherapy was not associated with 5-year DFS (HR 1.576, 95% CI 0.736–2.504; P = 0.082; **Figure 6A**); however, after stratification by our generated radiomics nomogram, 45.8% improvement of 5-year DFS were observed by additional chemotherapy in the high-risk group (HR 4.264, 95% CI 1.321–7.248; P = 0.008; **Figure 6B**), whereas no significant improvement of the 5-year DFS in the low-risk group (HR 0.565, 95% CI 0.281–3.132; P = 0.071; **Figure 6C**).

## Discussion

Due to its non-invasive advantages, medical imaging is often used in disease diagnosis, treatment, and the dynamic evaluation of therapeutic effects, especially for patients with cancer (16–19). Currently, traditional image analysis is qualitative, and there are large subjective differences that limit its clinical value (9, 20, 21). In recent years, medical imaging has achieved rapid development, especially with the advent of radiomics, which has enabled high-throughput information extraction from imaging features, and it is possible to quantify differences between tissues that cannot be observed by the naked eye (12, 20, 22). In this study, we used high-throughput methods to extract radiomics features and develop a radiomics signature, which can be used to predict DFS and the response of postoperation chemotherapy in patients with residual breast tumors after NAC. Using our prognostic radiomics signature classifier, the high-risk group exhibited a worse 5-year DFS rate (48%) than those in the low-risk group (89%). We also proved that our constructed radiomics nomogram, which combined the radiomic signature and clinical risk factors, has a better prediction performance than the radiomics signature alone. However, the benefit of additional chemotherapy after surgery in these patients remains unclear. In all enrolled patients, additional chemotherapy was not associated with 5-year DFS, which is consistent with previous studies. However, our study showed that high-risk group patients could get significant benefit from additional chemotherapy, whereas patients stratified as low-risk did not get any benefit.

We demonstrated that the radiomics signature, Ki-67 expression at surgery, and ALN status at surgery were outstanding clinical predictors of DFS in patients with residual breast tumors after NAC. The Ki-67 expression level in residual tumor tissues is a significant risk factor and a prognostic predictor of the chemotherapy response in patients who have residual tumors after the administration of NAC. Our results concur well with those of published studies (23–27). ALN status at surgery has been demonstrated to be a risk factor for 5-year DFS in non-pCR patients with BC, which is similar to the results of previous studies as well (28–31). Interestingly, four DFS-related radiomics features were selected in the current study, including one first-order features, one GLSZM features, and two GLCM features. The well-known radiomics features, entropy, was not included. Kurtosis, selected from one of the first-order features, was negatively associated with the risk of disease in this study. Many recent studies have particularly emphasized the significance of Kurtosis in colorectal, pancreatic, and breast cancers (16, 32, 33). The large area emphasis belongs to one of the features of GLSZM and is very suitable for quantifying the texture and heterogeneity of tumors because it considers the interaction between adjacent pixels (34, 35). Correlation feature shows the linear dependency of gray level values, and the maximum probability is the appearance of the most predominant pair of neighboring intensity values obtained from the GLCM features. These GLCM features reflect the texture heterogeneity of tumors in different aspects for they have different mathematical definitions.

Accordingly, we developed a nomogram based on these radiomics features for prediction of the DFS status and management of additional treatment strategies for each non-pCR patient with BC. The parameters of the nomogram can be easily obtained. For example, both Ki-67 expression at surgery and ALN status at surgery are conventional predictive factors and components of the TNM system in BC patients. In addition, the radiomics features could be extracted from breast tumor image *via* engineered hard-coded feature algorithms. In summary, our study demonstrated that the nomogram may serve as either a scoring system or a useful tool for chemotherapy response and prognostic prediction in non-pCR patients with BC, thus aiding physicians to rapidly evaluate the risk of relapse *via* a simple calculation method in the clinic.

Overall, our study has two strengths. First, this is the first study (to our knowledge) conducted to predict survival and postoperative chemotherapy response in invasive BC patients who received NAC and surgery using a radiomics signature. This study found that the radiomics nomogram can predict BC patient survival with a higher C-index and better calibration than the radiomics signature, with a higher C-index and better calibration. Second, because all radiomics features had different ranges, we standardized radiomics features values prior to the LASSO analysis, which achieved better predictive efficacy of the radiomics features. However, there were also several limitations in our study. First, our study included a small number of enrolled patients. Further studies in larger populations are needed, although we used the LASSO Cox method (10-fold cross-validation) to prevent overfitting. Second, a larger multicenter database that combines genomic and radiomics parameters has the potential to achieve a better performance of our current radiomics nomogram. Third, CT-based radiomics signature were used in the study, but the contrast resolution of the soft tissues was low in CT than MR imaging. Finally, we profiled the ROIs on the whole tumor area and calculated the radiomics predictors semi-automatically, which were time-consuming and laborious tasks. However, we believe that our research, mainly as a proof-of-concept study, has demonstrated the potential of the use of radiomics signatures in clinical practice. With the advent of commercially available software that offers automatic segmentation of tumors and automatic derivation of radiomics predictors, radiomics signatures are bound to be applied to daily clinical practice in the near future.

In conclusion, we observed a predictive radiomics signature might be a potential biomarker of risk stratification for DFS in invasive BC patients with non-pCR after NAC. Additionally, this study presents a radiomics nomogram that combined the radiomics signature and clinicopathological findings can assist in preoperative risk stratification, individualized predictions of recurrence and evaluate whether non-pCR patients will benefit from adjuvant chemotherapy after surgery. Therefore, our radiomics nomogram model may be potentially useful for personalized medicine and subsequently customize treatment strategies for BC patients with residual tumors after NAC.

## Data Availability Statement

All datasets generated for this study are included in the article/**Supplementary Material**.

## Ethics Statement

The studies involving human participants were reviewed and approved by the ethics committee of Sun Yat-sen University Cancer Center. The patients/participants provided their written informed consent to participate in this study.

## Author Contributions 

LZ, XJ, XHX, and SZ contributed to study design and development of methodology. WT collected the data. XHX, YW, and XJ analyzed the data. LZ, XMX, LL, XHX, XJ, and SZ wrote the manuscript. LL and XHX provided study supervision. All authors contributed to the article and approved the submitted version.

## Funding

This work is supported by the National Key R&D Program of China (2017YFC0112605), the National Natural Science Foundation of China (81302318, 81672598), the Science and Technology Planning Project of Guangdong Province (2017A020215163, 2016B090918066), and Guangzhou Municipal Science and Technology Project (201704020060, 201807010057).

## Conflict of Interest

The authors declare that the research was conducted in the absence of any commercial or financial relationships that could be construed as a potential conflict of interest.

## Abbreviations

ALN, axillary lymph nodes; AUC, area under the curve; BC, breast cancer; CT, computed tomography; DCA, decision curve analysis; DFS, disease-free survival; ER, estrogen receptor; FISH, fluorescence *in situ* hybridization; HER2, human epidermal growth factor receptor 2; IHC, immunohistochemistry; LASSO, least absolute shrinkage and selection operator; MRI, magnetic resonance imaging; NAC, neoadjuvant chemotherapy; pCR, pathological complete response; PR, progesterone receptor; ROIs, regions of interests.
