# Supplementary material for: The Impact of Preoperative Radiomics Signature on the Survival of Breast Cancer Patients With Residual Tumors After NAC
Source: Front Oncol. 2021 Feb 3;10:523327. doi: 10.3389/fonc.2020.523327 (PMC7888274; doi:10.3389/fonc.2020.523327)
Supplement: Supplementary file 1 [file DataSheet_1.docx]

**Supplementary materials:**


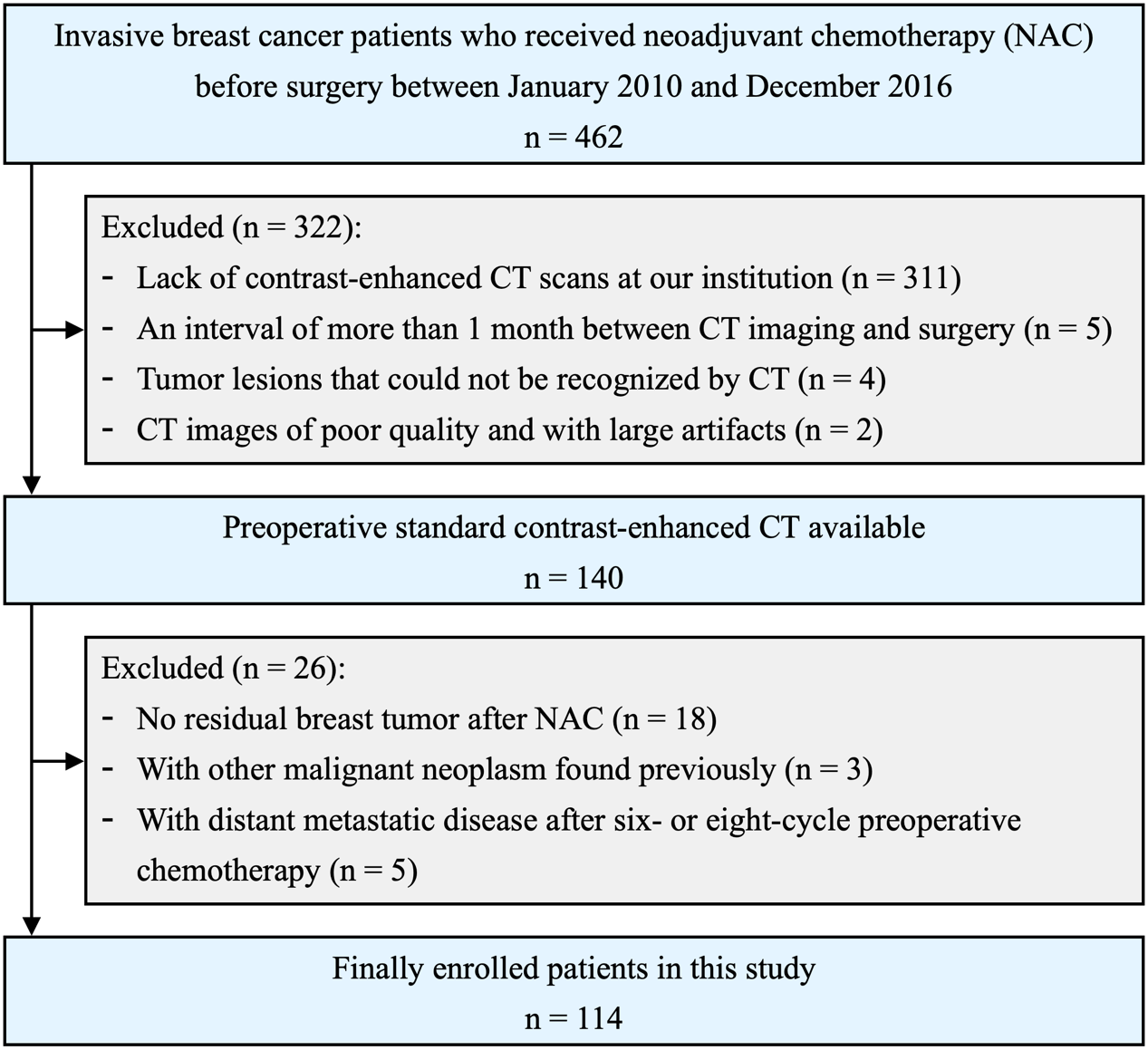


**Figure S1.** Flowchart of the enrolled patients.


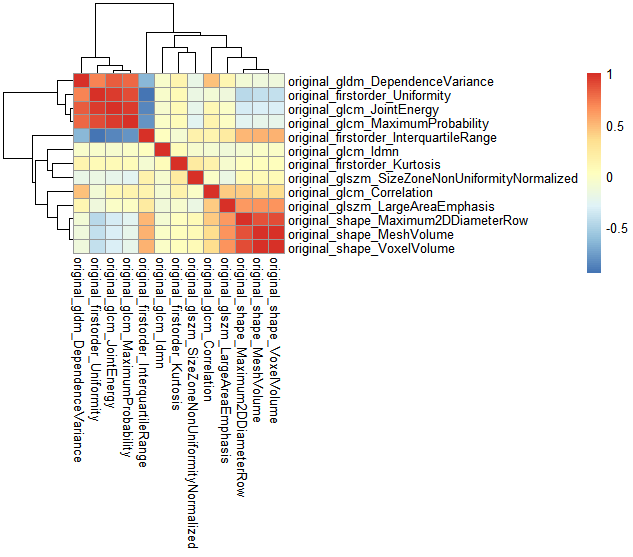


**Figure S2.** Hierarchical clustering shows the collinearity of 13 candidate radiomics features.


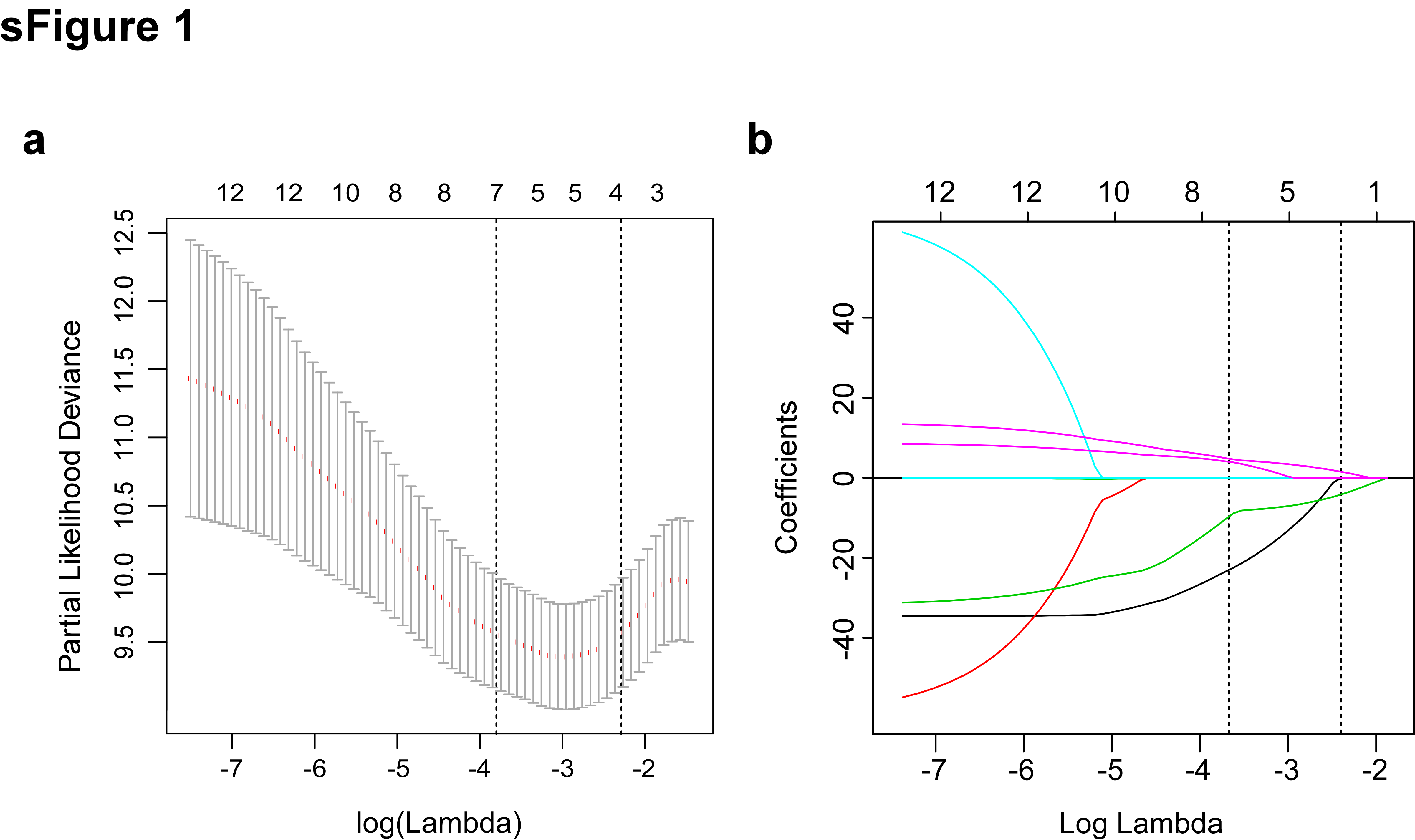


**Figure S3.** Construction of a radiomics signature. (a) LASSO algorithms was performed to determine DFS-related radiomics features and ultimately four optimal features were selected based on the training cohort. (b) LASSO coefficent profiles of the 13 DFS-status-related radiomics features.

**Table S1.** Clinicopathological characteristics of all patients in our study

| **Characteristics** | **Training cohort (n = 76)** | **Validation cohort (n = 38)** | ***P*-value** |
| --- | --- | --- | --- |
| Age, mean ± SD, years | 48.22 ± 9.447 | 49.03 ± 9.087 | 0.593 |
| Menopausal status |  |  | 0.684 |
| Pre | 47 (61.8) | 22(57.9) |  |
| Post | 29 (38.2) | 16(42.1) |  |
| Initial tumor status |  |  | 0.381 |
| T2 | 16 (21.1) | 11 (28.9) |  |
| T3 | 20 (26.3) | 6 (15.8) |  |
| T4 | 40 (52.6) | 21 (55.3) |  |
| Initial ALN status |  |  |  |
| Negative | 7 (9.2) | 4 (10.5) | 0.823 |
| Positive | 69 (90.8) | 34 (89.5) |  |
| Initial ER status |  |  | 0.888 |
| Negative | 25 (32.9) | 13 (34.2) |  |
| Positive | 51 (67.1) | 25 (65.8) |  |
| Initial PR status |  |  | 0.894 |
| Negative | 33 (43.4) | 17 (44.7) |  |
| Positive | 43 (56.6) | 21 (55.3) |  |
| Initial HER-2 status |  |  | 0.505 |
| Negative | 41 (53.9) | 23 (60.5) |  |
| Positive | 35 (46.1) | 15 (39.5) |  |
| Initial Ki-67 ( % ) | 35.66 ± 18.679 | 35.84 ± 20.253 | 0.766 |
| Tumor size at surgery |  |  | 0.868 |
| ≤ 2 cm | 27 (35.5) | 15 (39.5) |  |
| 2-5 cm | 38 (50.0) | 17 (44.7) |  |
| >5 cm | 11 (14.5) | 6 (15.8) |  |
| Grade at surgery |  |  | 1.0 |
| I/II | 64 (84.2) | 32 (84.2) |  |
| III | 12 (15.8) | 6 (15.8) |  |
| Vascular invasion at surgery |  |  | 0.722 |
| Absent | 64 (84.2) | 31 (81.6) |  |
| Present | 12 (15.8) | 7 (18.4) |  |
| ALN status at surgery |  |  | 0.393 |
| 0 | 16 (21.1) | 12 (31.6) |  |
| 1-3 | 28 (36.8) | 14 (36.8) |  |
| ≥4 | 32 (42.1) | 12 (31.6) |  |
| ER status at surgery |  |  | 1.0 |
| Negative | 28 (36.8) | 14 (36.8) |  |
| Positive | 48 (63.2) | 24 (63.2) |  |
| PR status at surgery |  |  | 0.691 |
| Negative | 41 (53.9) | 19 (50.0) |  |
| Positive | 35 (46.1) | 19 (50.0) |  |
| HER-2 status at surgery |  |  | 0.352 |
| Negative | 39 (51.3) | 23 (60.5) |  |
| Positive | 37 (48.7) | 15 (39.5) |  |
| Ki-67 at surgery (%) | 30.93 ± 25.345 | 27.50 ± 24.586 | 0.630 |
| Adjuvant chemotherapy |  |  | 0.891 |
| No | 47 (61.8) | 24 (63.2) |  |
| Yes | 29 (38.2) | 14 (36.8) |  |
| Adjuvant endocrine therapy |  |  | 0.781 |
| No | 26 (34.2) | 14 (36.8) |  |
| Yes | 50 (65.8) | 24 (63.2) |  |
| Radiomics signature | 0.16 ± 0.183 | 0.19 ± 0.20 | 0.536 |
| Event |  |  | 0.393 |
| Absent | 54 (71.1) | 24 (63.2) |  |
| Present | 22 (28.9) | 14 (36.8) |  |

ALN, axillary lymph node.
